# e-Health Literacy and Female Health Workers’ Readiness in Low-Resource Crisis Settings: The Mediating Role of Self-Efficacy

**DOI:** 10.3390/healthcare14213764

**Published:** 2026-11-04

**Authors:** Ashwani Panesar, Shashi Kant, Kiran Sood, Simon Grima

**Affiliations:** 1 Chitkara Business School, Chitkara University, Rajpura 140417, India; ashwani.panesar@chitkara.edu.in; 2 Department of Management, School of Business Administration and Management, DMI-St. John the Baptist University, Mangochi P.O. Box 406, Malawi; publications@dmisjbu.edu.mw; 3 Women Researchers Council (WRC), Azerbaijan State University of Economics (UNEC), Baku AZ 1001, Azerbaijan; 4 Department of Insurance and Risk Management, Faculty of Economics, Management and Accountancy, University of Malta, MSD2080 Msida, Malta; simon.grima@um.edu.mt; 5 Faculty of Economics and Social Sciences, University of Latvia, LV-1586 Riga, Latvia

**Keywords:** digital health technologies, digital competencies, technology adoption, behavioural preparedness, low-resource settings, perceived capability

## Abstract

**Highlights:**

**What are the main findings?**

- E-health literacy strengthens female health workers’ readiness to adopt and use digital health technologies.
- Self-efficacy plays a partial mediating role, highlighting the importance of confidence and perceived capability in translating e-health literacy into readiness.

**What are the implications of the main findings?**

- The findings help to implement practical, task-based and continuous e-health training for frontline FHWs by developing context-specific digital solutions for resource-constrained facilities.
- They support digital infrastructure, affordable access, ongoing capacity building and gender-responsive e-health programmes.

**Abstract:**

**Background/Objectives**: Low-resource economies face acute shortages of healthcare workers and digital health skills. This digital divide impedes the successful implementation of e-health technologies and quality healthcare services by female health workers. This study examines the relationship between e-health literacy and readiness to use digital health technologies among female health workers in Malawi, with self-efficacy mediating the relationship in a low-resource healthcare context. The study aims to generate evidence to inform targeted capacity-building interventions, strengthen health workers’ digital readiness, and support more accessible, effective, and equitable healthcare delivery. **Methods**: A cross-sectional quantitative research design was employed, using structured questionnaires administered to 251 female health workers. Direct and mediating relationships were examined using structural equation modelling (SEM). **Results:** Findings showed that e-health literacy positively influenced readiness (β = 0.731, t = 4.416, *p* < 0.001) and self-efficacy (β = 1.061, t = 7.814, *p* < 0.001) among female health workers. Self-efficacy also positively affected readiness (β = 0.274, t = 2.250, *p* = 0.024). Mediation analysis confirmed a significant indirect effect of e-health literacy on readiness through self-efficacy (β = 0.291, 95% CI [0.162, 0.432], *p* < 0.001), indicating partial mediation. **Conclusions**: Theoretically, the study highlights the interplay among social cognitive, technology readiness, and expectancy theories in the context of a low-resource crisis; practically, it offers evidence to inform the design of digital capacity-building interventions for healthcare workers.

## 1. Introduction

### 1.1. Background and Rationale

Health workers play a critical role in the healthcare ecosystem by improving health service coverage and enhancing the accessibility and quality of healthcare services for patients [1]. Recent reports highlight that the global health workforce comprised approximately 122.1 million workers across 20 occupational cadres in 2023 [2]. Despite significant workforce growth, major shortages persist across regions, particularly in sub-Saharan Africa, where health worker density remains comparatively low. The estimated additional workforce required to achieve an effective universal health coverage score of 80 out of 100 includes 7.1 million doctors, 23.9 million nurses and midwives, 1.8 million dentists, and 1.6 million pharmacists [2]. In developing and low-income countries, the mismatch between the number of health workers and growing population needs has led to shortages of quality services [3]. Increased migration of health workers from these countries to seek better employment opportunities has further worsened access to quality care.

Women make up 67% of the global health and social workforce, and WHO estimates a shortfall of nearly 11 million skilled health workers by 2030, mainly in lower-income countries [1]. Improving health literacy is essential to achieving the 2030 Agenda for Sustainable Development and transforming health systems to provide equitable care [4]. Internet penetration and access help healthcare workers provide information to patients [5]. Studies report that female health workers (FHWs) experience greater duality and role ambiguity than their male counterparts, which affects their self-efficacy [6] and, in the era of digital transformation [7], their psychological and behavioural readiness to adapt to new technologies.

E-health literacy (eHL) is defined as the ability to seek, find, understand, and appraise health-related information from electronic sources to address health problems [5,8].

A study involving healthcare professionals and students across diverse countries reported differences in attitudes towards eHealth, suggesting that acceptance may be shaped not only by access to digital technologies but also by prior experience, perceived competence, and the demands of technology use [9]. Healthcare professionals’ attitudes towards eHealth vary across settings. Their professional roles, prior exposure to digital systems, training opportunities, and the sociocultural and institutional environments in which they work may shape these attitudes [9,10]. FHWs tend to demonstrate greater eHL than their male counterparts [5,11]. Africa will face a shortage of approximately 6.1 million health workers by 2030 [3]. Malawi has fewer than five physicians per 100,000 people, and in 2018 it had only 149 health workers per 100,000 people, far below the WHO recommendation of 445 health workers [3]. This shortfall can be addressed by ensuring FHWs can understand, use, and assess health-related information through digital technologies [5].

A low-resource crisis refers to a state of emergency caused by a pandemic, natural disaster, or conflict in an environment lacking basic funding, medical facilities, or infrastructure. Malawi is a landlocked lower-income country in Southern Africa with a population of around 20.4 million in 2022. With 76 per cent of the country’s population living within an 8 km radius of a health facility, health care access remains limited in rural areas due to affordability and availability constraints [11]. Self-efficacy (SE), the belief in one’s capability to perform a particular behaviour, has proven to be an accurate predictor of performance in the adoption of healthcare technologies [12]. Studies have shown a relationship between an individual’s perception of effectiveness and psychological responses such as anxiety, which in turn influence SE through mutual interaction [12]. Occupational self-efficacy helps in improving FHWs’ psychological readiness [6].

Readiness (RD) is a multi-attribute concept encompassing psychological, behavioural, and cognitive components that underlie an individual’s ability to perform, adapt, and learn to accomplish tasks efficiently [13]. Psychological, behavioural, and cognitive readiness collectively depicts an individual’s mental and emotional preparedness, ability to act and adapt, and capacity to understand and manage complex situations effectively [13]. Malawi is a low-resource crisis country due to low per capita income, limited internet affordability, poor infrastructure, and high rates of HIV- and tuberculosis-related deaths [3]. eHealth readiness cannot be assumed to be uniform across countries or diverse healthcare settings. In low-resource contexts such as Malawi, local infrastructure, opportunities for digital training, and confidence in using digital systems may be particularly relevant to understanding readiness for eHealth adoption [10,14].

Evidence shows the importance of eHL for health care providers and identifies gaps in the evidence. A systematic review of 14 publications and 3666 healthcare providers found a mean eHL Scale (eHEALS) score of 27.8 to 31.7 (40 being the maximum score and 8 the minimum score) [15]. Specifically, nurses’ mean scores were 28.2 in South Korea, 29.9 in Turkey, and 31.7 in Iran and 30.7 for nurses and nursing assistants in Greece. Two studies found that 59% and 70% of health care workers in Ethiopia had high eHL. Further, the findings must be interpreted with caution, as the studies included various professional groups, used different measurement methods, and were not nationally representative of FHWs specifically [15].

Beyond individual capabilities, the digital health landscape can also affect health workers’ ability to gain and use eHL. Global Digital Health Monitor overview (2023) reported that the digital health maturity phase was 4 for high-income countries and phase 3 for low-income countries [16]. The indicators measure the maturity of the national DH system, not individual health workers’ skills, and should be used as contextual evidence rather than as measures of skills [16]. A qualitative study in Tanzania with 30 primary health care workers reported variation in digital health literacy by professional cadre, infrastructure, access to training, and organisational culture [10]. Despite these results, no available evidence directly compares eHL levels among FHWs in developed, developing, and resource-constrained countries.

Norman and Skinner (2006) conceptualised the Lily Model of eHL, which defines the skills required to find, understand, and use digital health information [8]. The model integrates six dimensions of literacy, namely traditional, information, health, scientific, media, and computer literacy. By conceptualising these constructs, eHL facilitates translating information into action and improves FHWs’ confidence [5,17].

### 1.2. Literature Review and Theoretical Background

Social Cognitive Theory (SCT) bridges behavioural and cognitive learning theories, focusing on thinking and symbolic processing amid rewards and behaviour [18]. All behaviours are also rooted in the belief that one has the power to make desired changes, so other factors serve as guides for behavioural change [19]. SCT posits that behaviour is shaped by the interaction of personal, behavioural, and environmental factors [20]. Individuals tend to develop SE through learning, persuasion, experience, and emotional arousal [7]. Higher SE enhances RD by strengthening individuals’ cognitive understanding, psychological confidence, and behavioural willingness to use new technologies [19,20]. Parasuraman (2000) conceptualised Technology Readiness to explain how optimism and innovativeness influence an individual’s willingness to adopt and use new technologies, while discomfort and insecurity inhibit it [21]. eHL provides health workers with strong information, computer, and media literacy to evaluate information for reliability [22]. SE supports this process by helping FHWs feel confident they can use eHealth applications successfully, further improving their comfort level [23]. eHL and strong SE improve FHW readiness aspects [22]. Cognitive, behavioural, and psychological factors help FHWs become more competent and confident in adopting new technology [21,22]. Vroom’s Expectancy Model explains motivation as a function of three beliefs: effort will improve performance, performance will lead to valued outcomes, and those outcomes matter to the individual [24]. Adoption of e-health tools depends not only on technical access but also on whether they believe they can use the system effectively, that it will improve their work, and that the benefits are worthwhile [25]. eHL strengthens the first link in the model because health workers with better digital skills are more confident that effort will translate into successful use of online health systems [24]. Readiness reflects whether they feel prepared with knowledge, training, and organisational support, which increases expectancy and reduces uncertainty [25]. eHL empowers FHWs to prepare for and engage with digital technologies [26,27], as they use digital records to interpret and assess patient health information. Improved information literacy among FHWs supports diagnosis and communication, augmenting cognitive readiness [28]. Their ability to use these tools facilitates decision-making to boost their confidence, a predictor of psychological readiness [29,30]. FHWs’ proficiency with digital devices makes them better able to navigate electronic health records, reducing barriers and increasing their willingness to adopt behavioural readiness [31,32]. Reliable online medical information and digital training modules further help them build trust in these platforms and reduce anxiety [17,33].

FHWs with strong computer skills were more confident completing routine tasks [23]; however, despite improved caregivers’ psychological health and SE [6,31,34], they still feel insecure and anxious about using technology [12,21,35]. FHWs’ SE also reflects their self-confidence and belief in their ability to cope with professional difficulties [36], improve decision-making [37], and enhance behavioural readiness [38,39,40]. FHWs with empathy and communication skills are more psychologically ready and competent to face any clinical situation [41], as they are more knowledgeable and experienced [36], improving health literacy [42,43]. FHWs working across diverse geographies have conflicts in communication, confidence, and teamwork skills which reduce their SE [44].

RD, integrated with professionalism and a strong sense of SE, plays a decisive role in developing FHWs’ performance [30,40]. FHWs’ confidence in their ability to perform the task efficiently provides the psychological basis for embracing innovation [35]. Further, FHWs with strong professional identity and commitment perceive themselves as being more competent and capable of handling complex situations [30]. Experiences related to role, challenges, and collaboration reinforce SE by confirming competence and building self-confidence [36]. FHW support in adopting eHL influences SE [27,31], as the internet is the most accessible source of health-related information [45,46]. FHWs with increased eHL are more likely to interpret this information more effectively [47], with technology playing a key role in building this capability [48,49]. eHL boosts technology readiness by increasing confidence in using digital tools to improve personal value and instrumentality, aligning with Vroom’s theory. SE influences FHWs’ perceptions and emotional responses to technology use [50], improving health literacy skills [22] to accomplish tasks efficiently [51]. The RD of FHWs to use electronic health records, keeping the strength of context in mind, improves the overall efficiency of the health system [52]. The transtheoretical model identifies individual cognitive change as key to health behaviour change, with SE playing a mediating role; improved cognitive skills and stronger SE drive eHL [51]. Studies have found that perceived competence, performance expectancy, and task confidence strengthen the association between eHL and RD [53,54]. The above discussion helps us to hypothesise that:

**H1.**  *e-Health Literacy has a positive and significant effect on Readiness.*

**H2.**  *Self-Efficacy has a positive and significant effect on Readiness.*

**H3.**  *e-Health Literacy has a positive and significant effect on Self-Efficacy.*

**H4.**  *Self-Efficacy mediates the relationship between e-Health Literacy and Readiness.*

### 1.3. Research Gap and Objectives

Most existing studies have focused on variables from the Technology Acceptance Model, Unified Theory of Acceptance, and Use of Technology while overlooking the interplay among RD, eHL, and SE [11,55]. Studies have also found that SE mediates the relationship between eHL and RD [55]. This study in Malawi highlights how adopting eHL among FHWs can improve readiness for better patient care. The study provides actionable insights into the competencies that need to be developed among FHWs. Bridging the SE gap can enhance healthcare service quality and promote greater RD among FHWs. Despite the growing literature on eHL and its relation to SE and RD, few studies have examined how healthcare workers’ readiness to adopt eHL influences SE in the Malawian context. Previous studies have explored the association between eHL and health-related behaviours [11]; however, limited attention has been given to the relationship between eHL and RD. FHWs need in-depth knowledge of eHL because low levels are associated with lower cognitive and psychological readiness, while improved access to online information enhances communication between caregivers and patients [28,55]. This is especially relevant in Malawi, where healthcare workers operate within strained services, and their digital adaptability, confidence, and cognitive skills play a crucial role in strengthening the overall healthcare system. Caregivers, particularly women, also shoulder substantial domestic responsibilities alongside their professional duties in Malawi due to the lack of essential care-supportive infrastructure [11]. Based on the above gaps, the study aims to answer four questions:

- What is the impact of e-health Literacy on Readiness?
- What is the impact of Self-Efficacy on Readiness?
- How does e-health literacy affect self-efficacy?
- How does self-efficacy mediate the relationship between e-health literacy and Readiness?

## 2. Materials and Methods

### 2.1. Study Design

The relationships between eHL, SE and RD are explored through a cross-sectional quantitative research design in this study. This study aims to test direct and mediation relationships using Structural Equation Modelling (SEM), which is appropriate for analysing latent variables and complex cause-and-effect relationships. The research design includes validation of the dimensional and mediational model to test the hypothesised relationships. Using a structured questionnaire, data were collected from FHWs.

### 2.2. Study Area

The study was carried out in the Central, Southern, Northern, and Eastern regions of Malawi, in the Districts of Lilongwe, Blantyre, Mzuzu, and Zomba, respectively. These sites were chosen to reflect variation in health care delivery and the state of digital health across the country. Data collection was carried out in the public health care system at various levels of health care delivery, e.g., central and district hospitals and health centres/clinics. The study was therefore conducted with health professionals in both urban and relatively resource-poor sites in Malawi. Structured questionnaires were used to collect data from the female health care workers in the selected study areas from May 2026 to July 2026.

### 2.3. Participants and Sampling

The respondents were female health care professionals with various roles, including nurses, clinical officers, doctors, midwives, and allied health staff. The sample is diverse in age, work experience, and facility type (district hospitals, central hospitals, and health centres/clinics). The study is conducted in the public healthcare system in low-resource and crisis environments, thus providing contextual relevance. The respondents were selected using the method of convenience sampling (non-probabilistic), which was done because of accessibility and willingness to participate. Given the operational and logistical limitations in crises, this was deemed the best approach, without employing several conflicting sampling strategies to ensure participation.

### 2.4. Measurement Instrument

A structured instrument adapted from the literature was used to measure the constructs. The questionnaire included constructs such as digital access, task confidence, perceived competence, performance expectancy, behavioural readiness, psychological readiness, cognitive readiness, user engagement, and digital literacy. Multiple items were used to assess each of the constructs, which were designed to cover the concept as thoroughly as possible. Items were rated on a 5-point Likert scale from strongly disagree to agree strongly. The instrument was created to be clear, relevant and appropriate for the healthcare context.

The validity and reliability of the measurement instrument were planned before hypothesis testing by applying standard SEM procedures. Cronbach’s alpha was used to assess internal consistency, composite reliability was measured using average variance extracted (AVE), and convergent validity was assessed using AVE. To examine discriminant validity, the Fornell–Larcker criterion and the HTMT ratio were used to ensure that constructs are empirically distinct.

### 2.5. Data Collection Procedure

The research team distributed questionnaires in person (offline) to healthcare facilities. Offline questionnaire distribution was selected to facilitate accessibility to FHWs working in diverse locations. Moreover, physical distribution enabled the research team to approach eligible FHWs at their workplaces and provide a consistent explanation of the study and its voluntary participation requirements. Informed consent, privacy, and anonymity were carefully observed during the data collection process. The questionnaires were distributed to 300 respondents; 268 were returned, and after screening for completeness and consistency, 251 valid questionnaires were used for data analysis.

### 2.6. Conceptual Framework

**Figure 1:**
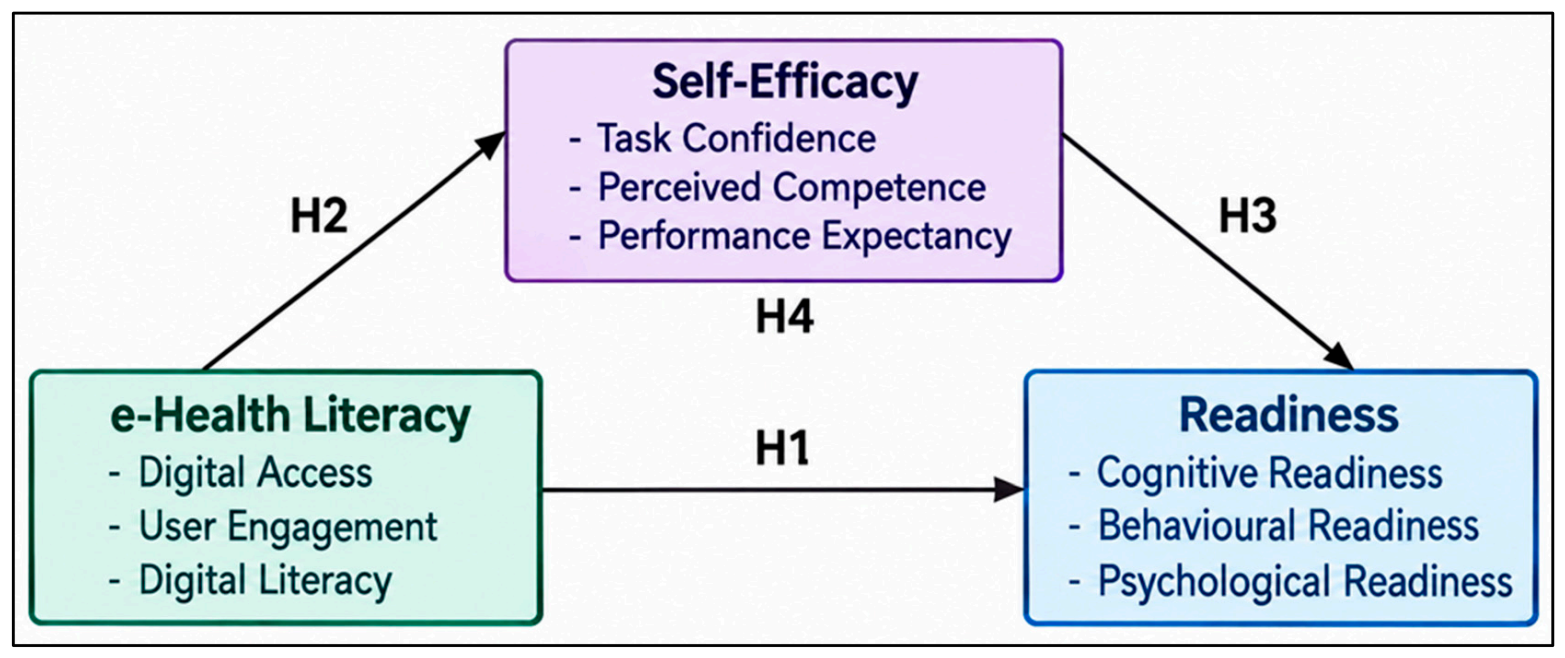
Proposed framework.

Figure 1 presents the study’s conceptual framework. It encompasses three variables.

The conceptual framework includes eHL (Digital access, User engagement, and Digital literacy) as the dependent variable; readiness (Cognitive, Behavioural, and Psychological) as the independent variable; and Self-Efficacy (Task confidence, Perceived competency, and Performance expectancy) as the mediating variable.

## 3. Results

### 3.1. Demographics

The sample’s demographic characteristics offer important implications for understanding the study’s results and for advancing e-health education strategies in low-resource crisis settings, as detailed in Table 1.

First, the study includes only FHWs, as is often the case in many low-resource settings where most frontline care workers (nursing, midwifery) are female. This not only suggests the value of gender-sensitive policy development but also limits the study’s generalizability to male health workers. It recommends that efforts to build e-health readiness consider gender-specific barriers to e-health, such as access to digital tools, workload, and sociocultural factors.

Second, given the range of qualifications among medical staff, such as medical practitioners, nurses, midwives, and allied health workers, the medical staff are likely to be multidisciplinary. However, the largest share is that of nurses and nurse-midwife technicians (38.2%), followed by clinical officers (20.7%). This means e-health education interventions should primarily target mid- and frontline care providers, the lifeline of care provision in crises. Training modules should therefore be based on skills and knowledge that are more practical, protocol-driven, and task-based, and not too specialised, in relation to digital competencies.

Third, the age distribution shows a relatively higher proportion of the workforce in the 25–34 age group (43.0%). This age group is generally pro-digital learning platforms, implying a positive context for e-health interventions to be scaled. However, the prevalence of older groups (36.2% at age 35 years and above) indicates that older people will need a more inclusive training approach that accounts for different digital literacy levels.

Fourth, most respondents had 5–10 years of work experience (38.2%), with a significant number having less than 5 years of work experience (29.5%). However, differences in SE in e-health between early-career and mid-career professionals point to inherent differences that may be influenced by recent training exposure and practical experience. It emphasises the need for constant professional development initiatives to build confidence and adaptability around digital health tools.

Finally, the cross-tabulation analysis of respondents by type of health facility (district hospital (41.4%), health centre/clinic (33.5%), and central hospital (25.1%)) reveals that most respondents work in low-resource, decentralised health facilities. This is especially relevant for infrastructure readiness, particularly in lower-quality facilities where internet and equipment may be less readily available or technically sound. So, e-health education activities should be designed to be low-bandwidth, offline, and context-specific.

In conclusion, the general conclusion that can be drawn is that e-health education interventions in emergencies must be gender responsive, level responsive, age inclusive, experience responsive and infrastructure responsive. These factors contribute to FHWs’ preparedness and the mediating effect of SE on the adoption of digital health innovations.

### 3.2. Measurement Model

**Figure 2:**
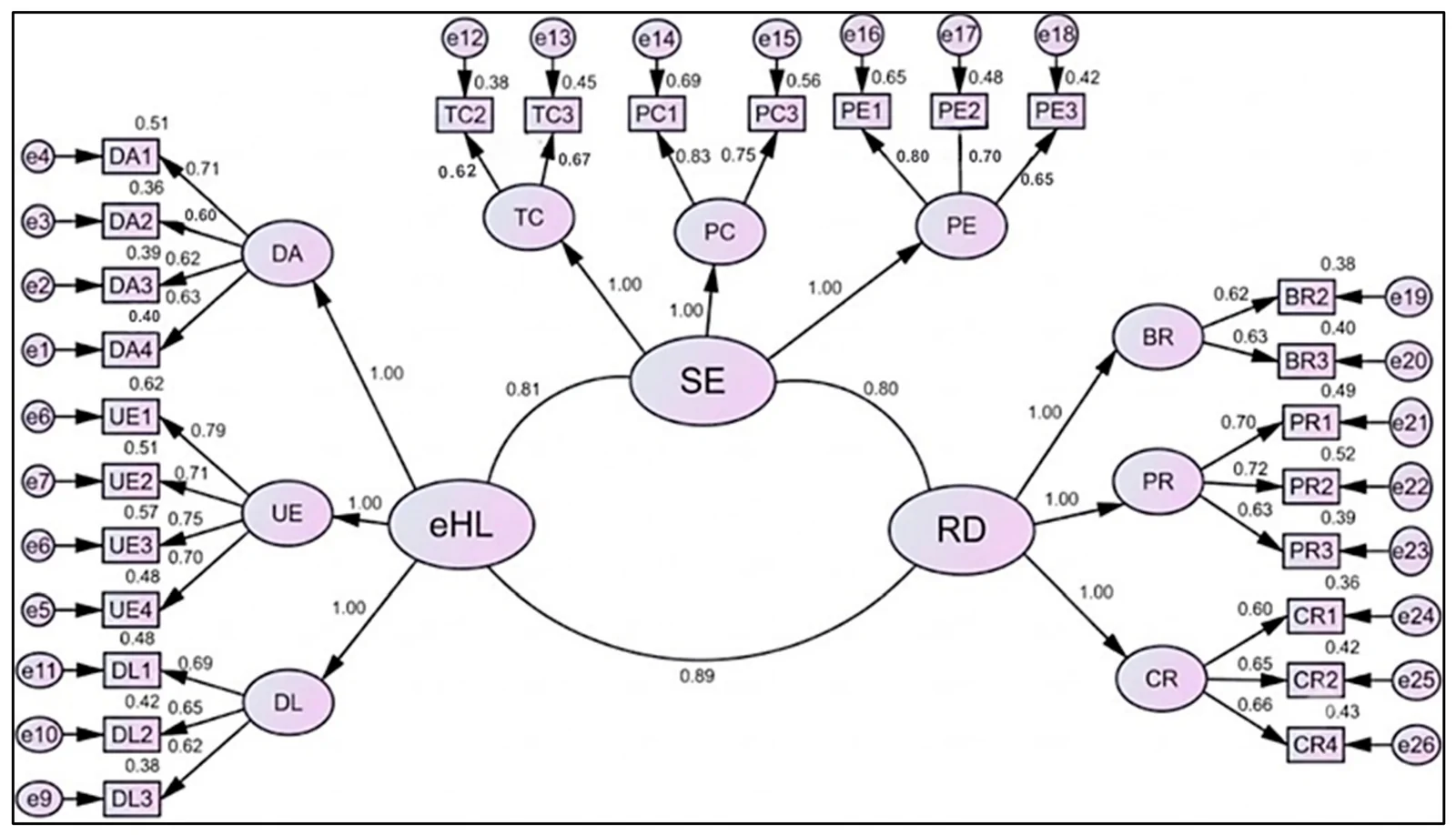
Confirmatory factor analysis.

Figure 2 presents the confirmatory factor analysis (CFA) model used to assess the measurement properties of the study’s constructs. It depicts the relationships between the observed measurement items and their respective latent constructs, providing a basis for evaluating construct reliability and validity before testing the structural model.

Table 2 shows acceptable reliability and validity for most constructs measuring e-health readiness, except for World Health Organisation health system readiness, which showed good results. Most factor loadings are >0.60, indicating acceptable construct representation, and all √AVE values are >0.60, indicating good convergent validity. Constructs with satisfactory reliability for most of the variables are composite reliability and Cronbach’s alpha, and constructs like task confidence (TC) under SE and behavioural readiness (BR) under readiness have relatively low reliability, which is suggested to be improved for future studies. Furthermore, there is no multicollinearity problem, as the VIF values do not exceed the critical threshold. Overall, the model is suitable for structural analysis and can be applied to understanding e-health readiness in low-resource crisis settings by employing factors such as digital literacy, perceived competence, and user engagement. Some constructs (e.g., TC and SP) had α values slightly below 0.70 but were retained because of their theoretical importance and adequate factor loadings in previous SEM studies.

### 3.3. Discriminant Validity

The Fornell and Larcker test results in Table 3 show that the constructs have discriminant validity, as the dimensions, digital access (DA), perceived competence (PC), and user engagement (UE), are empirically separable but moderately related. Hence, it substantiates the conceptual hierarchy of e-health readiness.

The HTMT ratios in Table 4 are mostly below the suggested threshold of 0.90, further supporting discriminant validity. A few values (e.g., UE with DL and PE) approach higher values, but they remain acceptable, as the associations are strong and theoretically meaningful, not redundant. We used both discriminant validity and the HTMT ratio to assess discriminant validity. The internal consistency of each construct was validated by square root of AVE values that were larger than its correlation within constructs, which established discriminant validity. In addition, all HTMT values were below the suggested threshold of 0.90, indicating that the constructs are distinct.

Overall, these results suggest that the constructs show good distinctiveness and reliability for structural model testing. This reinforces confidence in the mediating effect of SE and indicates that several aspects of e-health education uniquely impact FHWs’ preparedness in low-resource crisis environments.

### 3.4. Structural Model and Hypothesis Testing

**Figure 3:**
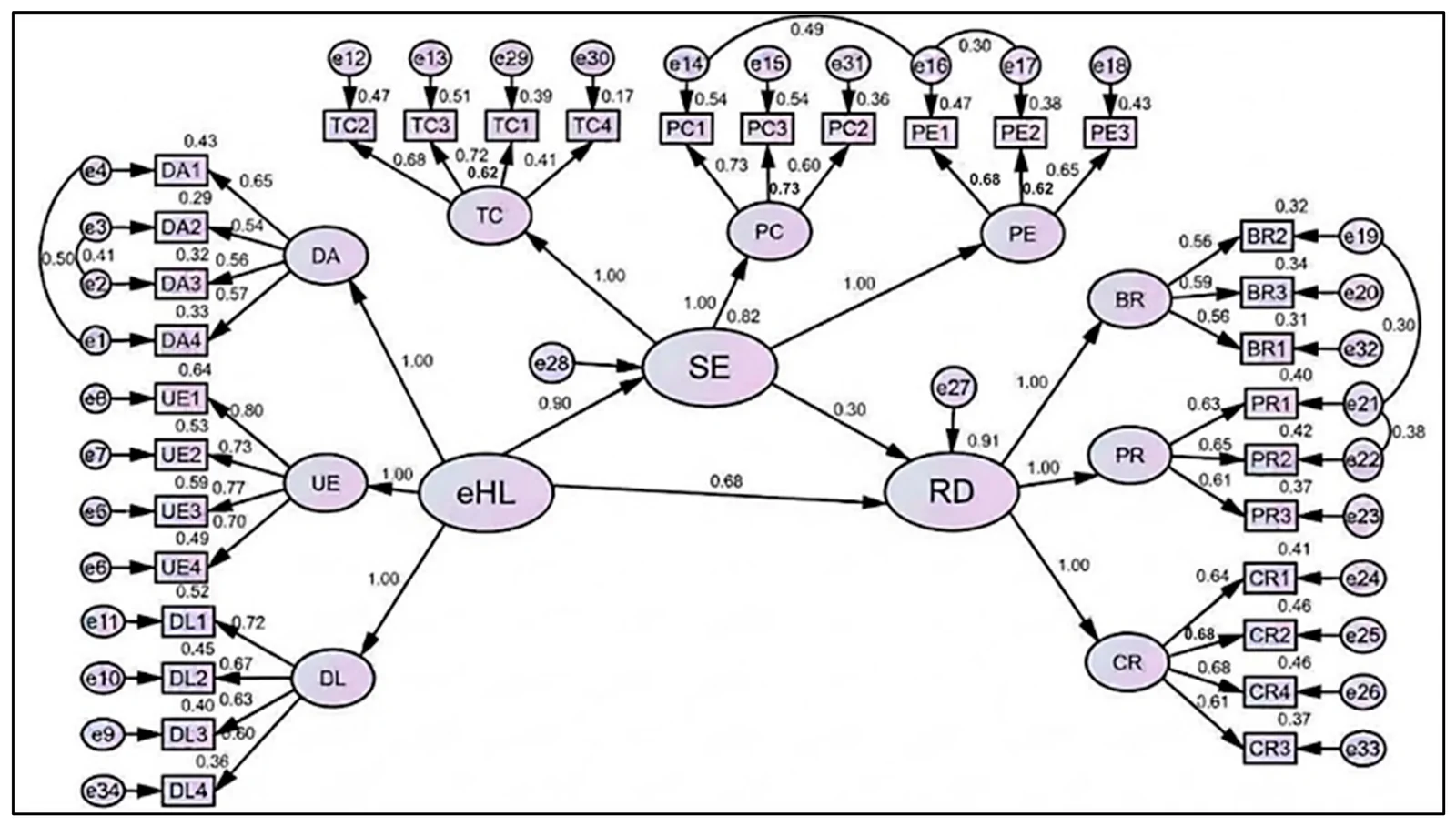
Structural equation model.

Figure 3 highlights structural relationships among eHL, SE, and RD among FHWs in low-resource crisis settings. The model examines the direct paths from eHL to RD, from eHL to SE, and from SE to RD. These paths represent the hypothesised relationships tested in the structural model, with corresponding path coefficients and significance levels reported in Table 5.

Table 5 indicates that eHL has a strong, statistically significant positive relationship with RD (β = 0.731, t = 4.416, *p* < 0.001), supporting H1. eHL also positively influences SE (β = 1.061, t = 7.814, *p* < 0.001), supporting H2. SE also has a positive, statistically significant relationship with RD (β = 0.274, t = 2.250, *p* = 0.024), supporting H3, although the effect is smaller. Collectively, the structural model suggests that eHL is an important factor associated with FHW readiness to use e-health technologies. At the same time, SE provides an additional pathway through which digital competencies contribute to their readiness.

### 3.5. Mediation Analysis

Mediation analysis results in Table 6 show that the relationship between e-HL and RD is partially mediated. This suggests that eHL may have a direct and indirect impact on RD via boosting individual confidence. Partial mediation indicates that other factors in the context or institution may also influence readiness. Overall, the findings suggest that combining skills training with SE-building approaches is an effective way to deliver e-health education. This two-pronged effort can enhance technical and psychological preparedness to implement e-health solutions in resource-limited settings.

Using the Sobel test shown in Table 7, the mediation of SE between eHL and readiness is significant (Z = 2.16, *p* < 0.05). This further supports the mediation effect revealed by bootstrapping, suggesting that the indirect route through SE is statistically significant. It reinforces the theoretical claim that psychological factors such as confidence significantly influence the ability to apply digital competencies in real situations during a crisis.

### 3.6. Model Fit

The model fit indices in Table 8 show that the proposed structural model has an overall acceptable to good fit. The key indicators, χ^2^/df, RMSEA, SRMR, IFI, and CFI, were within acceptable limits, indicating a recommended model fit. Some indices (e.g., GFI, NFI) are in acceptable (not excellent) ranges but are still in permissible ranges when used in applied research scenarios with complex models. These findings suggest a statistically valid and theoretically logical study model. This supports the validity of the relationships studied and aids the application of the model to understand e-health education and readiness among FHWs in low-resource crisis environments.

## 4. Discussion

This study explores the interrelationships between eHL SE and RD in 251 FHWs in Malawi. The results support the hypothesised relationships and suggest direct and indirect links between eHL and readiness through SE. These results build on the previous work by exploring the interrelationships in a resource-limited healthcare setting. The positive correlation between eHL and preparedness (β = 0.731, t = 4.416, *p* < 0.001) suggests that the more digital health literacy a person has, the more prepared they are to use e-health technologies. The results agree with the Lily Model, which focuses on the ability to access, understand and assess EHI [8]. It also confirms past research findings on technology readiness [27,46] and healthcare providers’ readiness to use electronic health records [31,32]. This is expanded to the case of female health workers in Malawi, where resources may limit technology uptake. However, digital competence requires strong infrastructure, reliable internet connectivity, and organisational support to be implemented effectively [10,16,20,48,53].

The positive relationship between e-HL and SE (β = 1.061, *p* < 0.001) means that digital health competencies are associated with FHWs’ confidence in using this technology. The findings are in line with SCT, which identifies SE as a key personal belief shaped by learning and experience [12,18]. It also embodies concepts of expectancy theory, which states that confidence is built when people believe they can attain better performance results when they work harder. Previous studies have also examined the relationship between eHL and SE among the caregivers and other populations [30,33]. This relationship also suggests that digital health training should provide opportunities for practical application, allowing FHWs to build confidence through experience rather than knowledge alone [35,56].

SE was positively associated with RD (β = 0.274, *p* < 0.05), suggesting that SE partially influenced FHWs’ preparedness to use digital tools, although the effect was smaller. These findings align with SCT, which highlights SE as a factor in improving technology readiness among pharmacy students and readiness for electronic health record adoption among health providers [23,31] and nurses [37]. The smaller effect suggests that confidence alone may not fully explain preparedness to use digital tools. Therefore, organisational and infrastructure support should be considered alongside individual capabilities [48,52].

SE acts as a mediator and is identified as an indirect relationship between eHL and RD (Indirect effect = 0.291, *p* < 0.001; Sobel Z = 2.16), indicating partial mediation. These findings align with previous studies showing that SE mediates the relationship between eHL and RD [55] and between eHL and health-related behaviours [51,57]. The remaining direct relationship suggests that SE does not fully account for the association between eHL and RD. This study contributes at both the theoretical and contextual levels by identifying a dual pathway (direct and indirect) in a low-resource crisis context, unlike studies that have focused so far on direct effects.

The results have implications for developing digital health capacities. The focus should be on task-based training, application, and ongoing professional development in a healthcare environment. When designing training, consider professional duties, age, and prior digital experience. For facilities with limited connectivity, low-bandwidth platforms and offline learning resources can improve accessibility. Investing in reliable equipment, affordable digital access, and sustained institutional support is also crucial. The measures may support the transfer of competencies into readiness for specific tasks but may not presume that the need for structural measures can be met through training.

## 5. Conclusions

The results indicate that eHL positively influences readiness both directly and indirectly through SE. Increased access, engagement, and digital literacy increase the confidence of FHWs in low-resource settings, thus boosting their readiness to use e-health systems. The direct relationship between eHL and RD is strong, while SE’s mediating role highlights psychological readiness. The study is unique because it combines SCT, ET, UTAUT, and the Lily model into a single framework specifically for low-resource crisis contexts. It highlights how RD is influenced by digital skills and digital confidence, an area of research mainly concerned with low-resource settings. In practice, stakeholders such as hospitals, educational institutions, and policymakers need to prioritise consistent, task-based digital health training to improve competency in an evolving technological world. This can be made possible by improving existing digital infrastructure and context-appropriate solutions such as digitisation and digitalisation.

## 6. Implications

Theoretically, this study builds on SCT, expectancy theory, UTAUT, and the Lily model of eHL, which are extrapolated to low-resource crisis environments. The results support the hypotheses, indicating that e-HL positively predicts RD to act and that this effect occurs both directly and indirectly, via the mediating role of SE, as suggested by SCT. Moreover, the findings align with Expectancy Theory and UTAUT, which suggest that perceived capability and performance expectancy relate to adoption. Furthermore, the study demonstrates the influence of resource constraints on digital readiness and the contributions of various stakeholders, including policymakers and healthcare institutions, in enabling access to, training in, and support for the D2L system from an Institutional Theory perspective. Unlike previous research in developed settings, this study shows the need for capability aspects (SE) in a constrained setting and fills this gap in the context.

## 7. Study Limits and Future Work

This study has several limitations that need to be recognised. First, the study is self-reported, so common method bias and social desirability effects may be present. Second, the study involved a sample of 251 FHWs in Malawi, and the findings may not be fully generalised to all healthcare workers or other healthcare settings. We recommend that future studies use larger, more diverse samples and triangulation methods, including objective performance measures or institutional records alongside survey data, to increase the validity of the findings. Second, focusing on FHWs and using purposive sampling in low-resource crisis settings may limit generalizability. Yet this context is a strength and boundary condition in that the findings are pertinent to resource-limited settings in which digital readiness is the most important. Further studies in different environments (developed or comparative) could investigate the model’s generalizability.

Lastly, although the study does examine SE as a mediator, other mediators could be explored and examined. Future research could include factors such as institutional support, training effectiveness, organisational culture, and leadership influence to deepen understanding of eHL adoption. The addition of these variables to the model can contribute to reinforcing theoretical and practical understanding of digital health readiness.

## Figures and Tables

**Table 1:** Descriptive of Demographics.

Variable	Category	N	%
Gender	Female	251	100.0
Medical Degree Type	Medical Doctor	38	15.1
	Clinical Officer	52	20.7
	Registered Nurse/Nurse Midwife Technician (NMT)	96	38.2
	Midwife (Diploma/Degree)	28	11.2
	Public Health Officer	21	8.4
	Allied Health (Lab, Pharmacy, etc.)	16	6.4
Age Group (in years)	Below 25	52	20.7
	25–34	108	43.0
	35–44	56	22.3
	45 and above	35	13.9
Work Experience	Below 5 years	74	29.5
	5–10 years	96	38.2
	11–15 years	51	20.3
	Above 15 years	30	12.0
Health Facility Type	District Hospital	104	41.4
	Central Hospital	63	25.1
	Health Centre/Clinic	84	33.5
Total Respondents	All participants	251	100.0

**Table 2:** Measurement Model Analysis.

Construct	Item	FL	√AVE	CR (CF)	α (Cronbach)	VIF
DA	DA1	0.71				
	DA2	0.60				
	DA3	0.62				
	DA4	0.63	0.641	0.735	0.73	2.31
TC	TC2	0.62				
	TC3	0.67	0.646	0.588	0.56	2.08
PC	PC1	0.83				
	PC3	0.75	0.791	0.768	0.76	2.54
PE	PE1	0.80				
	PE2	0.70				
	PE3	0.65	0.720	0.762	0.75	2.47
BR	BR2	0.62				
	BR3	0.63	0.633	0.570	0.55	1.96
PR	PR1	0.70				
	PR2	0.72				
	PR3	0.63	0.684	0.724	0.72	2.63
CR	CR1	0.60				
	CR2	0.65				
	CR4	0.66	0.637	0.671	0.67	2.41
UE	UE1	0.79				
	UE2	0.71				
	UE3	0.75				
	UE4	0.70	0.738	0.827	0.83	2.72
DL	DL1	0.69				
	DL2	0.65				
	DL3	0.62	0.654	0.691	0.69	2.19

**Table 3:** Correlation matrix with Fornell–Larcker criterion.

Construct	DA	TC	PC	PE	BR	PR	CR	UE	DL
DA	0.641								
TC	0.52	0.646							
PC	0.48	0.55	0.791						
PE	0.50	0.57	0.66	0.720					
BR	0.46	0.49	0.53	0.55	0.633				
PR	0.44	0.50	0.56	0.58	0.61	0.684			
CR	0.42	0.47	0.52	0.54	0.59	0.63	0.637		
UE	0.60	0.58	0.62	0.65	0.57	0.59	0.55	0.738	
DL	0.57	0.54	0.58	0.60	0.52	0.55	0.53	0.66	0.654

**Table 4:** HTMT ratio.

Construct	DA	TC	PC	PE	BR	PR	CR	UE	DL
DA	—								
TC	0.71	—							
PC	0.68	0.74	—						
PE	0.69	0.76	0.85	—					
BR	0.65	0.70	0.73	0.75	—				
PR	0.63	0.71	0.78	0.80	0.84	—			
CR	0.61	0.68	0.72	0.74	0.82	0.87	—		
UE	0.82	0.79	0.83	0.86	0.78	0.80	0.76	—	
DL	0.78	0.75	0.79	0.82	0.73	0.77	0.74	0.88	—

**Table 5:** Hypothesis check.

H	Path	Direct Effect (β)	t-Value	*p*-Value	f^2^	Effect Size
H1	eHL → RD	0.731	4.416	0.000	0.42	Large
H2	eHL → SE	1.061	7.814	0.000	0.67	Large
H3	SE → RD	0.274	2.250	0.024	0.08	Small

**Table 6:** Mediation bootstrapping.

H	Path	Direct Effect (β)	Indirect Effect (β)	Total Effect (β)	t-Value	*p*-Value	95% CI (LL)	95% CI (UL)	Decision	Mediation Type
H5	eHL → SE → RD	0.731	0.291	1.022	4.27	0	0.162	0.432	Recognize	Partial

**Table 7:** Sobel mediation test.

Path	a (eHL → SE)	b (SE → RD)	Sa	Sb	Sobel Z-Value	*p*-Value	Decision
eHL → SE → RD	1.061	0.274	0.136	0.122	2.16	0.031	Significant

**Table 8:** Model fitness.

Class	Index	Value	Clarification
Absolute Fit	χ^2^ (CMIN)	845.258	Substantial
	Df	455	—
	χ^2^/df	1.858	Good fit
	RMR	0.072	Adequate
	GFI	0.897	Suitable
	AGFI	0.882	Suitable
Incremental Fit	NFI	0.895	Satisfactory
	RFI	0.892	Acceptable
	IFI	0.906	Good fit
	TLI	0.900	Suitable
	CFI	0.905	Decent fit
Parsimony Fit	PNFI	0.749	Decent
	PCFI	0.830	Fine
Error (Approx.)	RMSEA	0.059	Better fit
	SRMR	0.072	Fine fit

## Data Availability

The raw data supporting the conclusions of this article will be made available by the authors on request.

## Abbreviations

The following abbreviations are used in this manuscript:

eHL	Electronic Health Literacy
FHW	Female Health Worker
SCT	Social Cognitive Theory
SE	Self-Efficacy
RD	Readiness
DA	Digital Access
TC	Task Confidence
PC	Perceived Competence
PE	Performance Expectancy
BR	Behavioural Readiness
PR	Psychological Readiness
CR	Cognitive Readiness
UE	User Engagement
DL	Digital Literacy
